# Nutritional Indices for Assessing Fatty Acids: A Mini-Review

**DOI:** 10.3390/ijms21165695

**Published:** 2020-08-08

**Authors:** Jiapeng Chen, Hongbing Liu

**Affiliations:** 1 Key Laboratory of Marine Drugs, Chinese Ministry of Education, School of Medicine and Pharmacy, Ocean University of China, Qingdao 266003, China; 21180831069@stu.ouc.edu.cn; 2 Laboratory for Marine Drugs and Bioproducts, Pilot National Laboratory for Marine Science and Technology (Qingdao), Qingdao 266237, China

**Keywords:** fatty acids, nutritional indices, human health

## Abstract

Dietary fats are generally fatty acids that may play positive or negative roles in the prevention and treatment of diseases. In nature, fatty acids occur in the form of mixtures of saturated fatty acid (SFA), monounsaturated fatty acid (MUFA), and polyunsaturated fatty acid (PUFA), so their nutritional and/or medicinal values must be determined. Herein, we do not consider the classic indices, such as ∑SFA, ∑MUFA, ∑PUFA, ∑n-6 PUFA, ∑n-3 PUFA, and n-6 PUFA/n-3 PUFA; instead, we summarize and review the definitions, implications, and applications of indices used in recent years, including the PUFA/SFA, index of atherogenicity (IA), the index of thrombogenicity (IT), the hypocholesterolemic/hypercholesterolemic ratio (HH), the health-promoting index (HPI), the unsaturation index (UI), the sum of eicosapentaenoic acid and docosahexaenoic acid (EPA + DHA), fish lipid quality/flesh lipid quality (FLQ), the linoleic acid/α-linolenic acid (LA/ALA) ratio, and *trans* fatty acid (TFA). Of these nutritional indices, IA and IT are the most commonly used to assess the composition of fatty acids as they outline significant implications and provide clear evidence. EPA + DHA is commonly used to assess the nutritional quality of marine animal products. All indices have their advantages and disadvantages; hence, a rational choice of which to use is critical.

## 1. Introduction

Fatty acids (FAs) are organic acids with at least one carboxyl (–C(=O)OH, –COOH, or –CO_2_H) group and a long carbon chain whose links can be double bonds, as in unsaturated fatty acids, or single bonds, as in saturated fatty acids. FAs are generally derived from triglycerides and phospholipids, and are the main components of dietary fats. Most naturally occurring FAs have an unbranched chain of an even number (4–28) of carbon atoms. According to the number of double bonds, the FA catalogue includes saturated fatty acids (SFAs), monounsaturated fatty acid (MUFA), and polyunsaturated fatty acid (PUFA).

FAs are distributed to cells where they serve as fuel for muscular contraction and general metabolism. As biological compounds, FAs play critical roles in human metabolism, health, and disease. Epidemiological studies and clinical trials showed that fatty acids are associated with cardiovascular diseases [1,2,3,4,5], neurological diseases [6,7,8,9], non-alcoholic fatty liver disease [10,11,12,13], allergic diseases [14,15,16], and so on. Evidence from metabolomics experiments indicates that they participate in the metabolic pathways of related diseases [8,17,18,19,20,21,22,23,24,25,26]. For example, the free FA profile was found to be altered in both leukemia and pre-leukemic diseases, particularly C14:0, C16:0, and C18:0 [26].

FAs play positive or negative roles in the prevention and treatment of diseases. For example, SFAs may increase the risk of developing multiple sclerosis (MS) as well as disease progression, whereas PUFAs may have beneficial effects in MS patients [7]. As another example, some essential FA metabolites may exert health effects such as anti-inflammatory and neuroprotection effects, but they can also produce negative effects such as inflammation, necrosis promoters, and atherosclerosis. In general, FAs are obtained from various dietary sources that possess characteristic FA composition and consequently influence health outcome. From this perspective, the FA composition should be assessed to determine their nutritional and/or medicinal value, especially in fatty-acid-rich foods, food supplements, and herb-based medicines. 

In this mini-review, we collated the literature related to fatty acid profile analysis that was published in recent decades since 2000 to understand the implications and applications of various nutritional indices. We did not consider the classic indices such as ∑SFA, ∑MUFA, ∑PUFA, ∑n-6 PUFA, ∑n-3 PUFA, and n-6 PUFA/n-3 PUFA. The present review may help researchers to evaluate the nutritional value of fatty acids and to explore their potential usage in disease prevention and treatment. It may also help newcomers to the field of fatty acid profile analysis to quickly and accurately select appropriate indices.

## 2. Nutritional Indices

In this review, we screened articles and summarized the nutritional indices. The results are shown in Table 1. 

### 2.1. Polyunsaturated Fatty Acid/Saturated Fatty Acid (PUFA/SFA)

PUFA/SFA is an index normally used to assess the impact of diet on cardiovascular health (CVH). It hypothesizes that all PUFAs in the diet can depress low-density lipoprotein cholesterol (LDL-C) and lower levels of serum cholesterol, whereas all SFAs contribute to high levels of serum cholesterol. Thus, the higher this ratio, the more positive the effect. 

All that was missing was MUFA. According to Dietschy’s study on dietary FAs and their regulation of plasma LDL-C concentrations in 1998, C18:1 n-9 *cis* (oleic acid), the most common MUFA in dietary food increases the activity of low-density lipoprotein receptors (LDLRs) and decreases the cholesterol concentration in serum [111]. Not all molecular species of SFAs contribute equally to serum cholesterol. C12:0, C14:0, and C16:0 can increase the cholesterol concentration in serum by inhibiting the activity of LDLRs; C4:0, C6:0, C8:0, and C10:0 were rapidly oxidized to acetyl-CoA in the liver and could not affect the activity of LDLRs, and C18:0 appeared to be biologically neutral and have no effect on circulating LDL-C levels [111].

Notably, not all of the main classes of PUFA positively affect the prevention of cardiovascular disease (CVD). Short-term supplementation with docosahexaenoic acid (DHA)-rich fish oil may modulate the activity of peroxisome proliferators-activated receptor-gamma (PPAR-γ) to protect the cardiovascular system from the unhealthy effects of atherosclerotic lesions [4]. Recent clinical trials support the view that supplementation with eicosapentaenoic acid (EPA) can reduce plasma triglyceride (TG) levels and activate anti-inflammatory, anti-thrombotic, and other mechanisms to prevent atherosclerosis (AS) [2]. However, a narrative review that collated the available data showed that dietary intake of linoleic acid (LA, C18:2 n-6) is inversely correlated with CVD; however, further research is needed to clarify the underlying mechanisms [3].

PUFA/SFA is the most commonly used index for evaluating the nutritional value of dietary foods such as of seaweed (0.42–2.12, except for *Gracilaria changii*), meat (0.11–2.042), fish (0.50–1.62), shellfish (0.20–2.10), and dietary products (0.02–0.175). Chan and Matanjun determined the FA profiles of red seaweed *Gracilaria changii* a mangrove area of Malaysia, and used PUFA/SFA to assess the nutritional quality, finding a value of 6.96 ± 0.98 [28]. PUFA/SFA of chicken is in the range of 0.308 to 2.042 for different dietary treatments [48]. Fernandes et al. compared the FA profile of four species of Brazilian fish, and they used the PUFA/SFA as one of the nutritional quality indices, reporting values between 1.09 to 1.47 [36]. Detailed information about the literature related to PUFA/SFA is shown in Table 2.

### 2.2. Index of Atherogenicity (IA)

The index of atherogenicity (IA) was developed by Ulbritcht and Southgate in 1991, and characterizes the atherogenic potential of FA [112]. As the PUFA/SFA ratio is too general and unsuitable for assessing the atherogenicity of foods, Ulbritcht and Southgate proposed a new index, IA, based on PUFA/SFA considering the available evidence, and then checked whether the resulting values were in accordance. The formula for calculating IA is:

(1) IA =[C12:0+(4 × C14:0)+ C16:0]/ΣUFA

The IA indicates the relationship between the sum of SFAs and the sum of unsaturated fatty acids (UFAs). The main classes of SFAs, which include C12:0, C14:0, and C16:0, with the exception of C18:0, are considered pro-atherogenic (they favor the adhesion of lipids to cells of the circulatory and immunological systems) [67,68,113]. UFAs are considered to be anti-atherogenic as they inhibit the accumulation of plaque and reduce the levels of phospholipids, cholesterol, and esterified fatty acids [68,113]. Therefore, the consumption of foods or products with a lower IA can reduce the levels of total cholesterol and LDL-C in human blood plasma [85]. 

Although the IA is more reasonable than the simple PUFA/SFA ratio for assessing the degree of atherogenicity, there are still some imperfections in the proposed IA formula, which were pointed out by Ulbritcht and Southgate. First, stearic acid (C18:0) should appear in the denominator if sufficient evidence shows that it can reduce the level of LDL-C in human blood plasma in the future. Second, not all PUFAs should be weighed equally. Third, the impact of *trans* fatty acids was not considered due to conflicting evidence [112].

The IA has been used widely for evaluating seaweeds, crops, meat, fish, dairy products, etc. Nantapo et al. analyzed the fatty acid composition of milk during different stages of lactation and found that milk with lower IA is important, and the IA ranges from 4.08 to 5.13 in different stages of lactation [56]. Akintola investigated the techniques of smoking and sun drying to understand the nutritional quality of southern pink shrimp (*Penaeus notialis*) using the IA as an index, and reported values of 0.71 to 0.82 [64]. 

Detailed information about the literature related to the IA is shown in Table 3. For seaweeds, the species may be the main factor influencing the IA value, which ranges from 0.03 to 3.58. The value ranges from 0.084 to 0.55 for crops, 0.21 to 1.41 for fish, and 0.165 to 1.32 for meat. For dairy products, the value ranges from 1.42 to 5.13. For ruminants, dietary treatment is the main factor influencing the IA.

### 2.3. Index of Thrombogenicity (IT)

The index of thrombogenicity (IT) was developed by Ulbritcht and Southgate [112] together with IA in 1991. The formula is:

(2) IT =(C14:0+ C16:0+ C18:0)/[(0.5×ΣMUFA)+(0.5×Σn−6 PUFA)+(3×Σn−3 PUFA)+(n−3 / n−6)]

The IT characterizes the thrombogenic potential of FAs, indicating the tendency to form clots in blood vessels and provides the contribution of different FAs, which denotes the relationship between the pro-thrombogenic FAs (C12:0, C14:0, and C16:0) and the anti-thrombogenic FAs (MUFAs and the n-3 and n-6 families) [112]. Therefore, the consumption of foods or products with a lower IT is beneficial for CVH. The IT has been used in many fatty acid composition studies to assess the degree of thrombogenicity. As with the IA formula, the proposed IT formula should be modified as our understanding of MUFA and *trans* fatty acids increases. 

The IT has been used in many FA composition studies to assess the degree of thrombogenicity. Chen et al. conducted comparative studies on the fatty acid profiles of four different Chinese medicinal *Sargassum* seaweeds, where the IT was used as one of the nutritional indices to evaluate the potential effects of four *Sargassum* on CVH. The results showed that the IT was between 0.46 and 1.60 [29]. Calabrò et al. compared the fatty acid profile of three cultivars of *Lupinus albus* (Lutteur, Lublanca, and Multitalia) and the IT was used due to the correlation between fatty acids and human health [30]. 

Detailed information of the literature related to the IT is provided in Table 4. For seaweeds, the value ranges from 0.04 to 2.94 with the exception of *Gracilaria salicornia,* which had an IT value of 5.75 [27]. The ranges of IT values for crops, fish, meat, and dairy products are 0.139–0.56, 0.14–0.87, 0.288–1.694, and 0.39–5.04, respectively. 

In brief, both the IA and the IT can be used to assess the potential effects of FA composition on CVH. A FA composition with a lower IA and IT has a better nutritional quality, and its consumption may reduce the risk of coronary heart disease (CHD), but no organization has yet provided the recommended values for the IA and IT. As our comprehensive understanding of the function of FA molecular species deepens, the accuracies of the IA and IT formulas are expected to increase, which might be modified by taking advantage of the massive amount of available data and advanced computer technology.

### 2.4. Hypocholesterolemic/Hypercholesterolemic (HH) Ratio

The hypocholesterolemic/hypercholesterolemic (HH) ratio is an index used in the FA profile of lamb meat first proposed by Santos-Silva et al. in 2002 [91]. Due to the high proportion of SFA, the PUFA/SFA is normally low in lambs, so Santos-Silva et al. developed the HH as a new index to assess the effect of FA composition on cholesterol.

Basic on research about dietary FA and the regulation of plasma LDL-C [111], the HH characterizes the relationship between hypocholesterolemic fatty acid (*cis*-C18:1 and PUFA) and hypercholesterolemic FA. Because there was no C12:0 detected in the lambs, Santos-Silva et al. concluded that the formula only includes C14:0 and C16:0 in hypercholesterolemic FA. Later, Mierliță optimized the formula by adding the C12:0 in hypercholesterolemic FA during the studies of sheep milk [55]. The formula is:

(3) HH =(cis−C18:1+ ΣPUFA)/(C12:0+ C14:0+ C16:0)

Compared with the PUFA/SFA ratio, the HH ratio may more accurately reflect the effect of the FA composition on CVD. The HH ratio has certain limitations. Similar to the IA and IT, the HH might include more kinds of fatty acids such as other molecular species of MUFA and different weights can be assigned to different molecular FA species.

The HH was first used in research on ruminants [46,77,91], which was subsequently extended to dairy products [54,55,78,86,87], marine products [34,36,37,38,39,72,90], and other fields [47,48,52,62]. Paiva et al. selected four Azorean macroalgae and used the HH as one of the indices to evaluate their nutritional and health promoting aspects, and found that the HH value ranges from 1.26 to 2.09 [90]. Ratusz et al. analyzed the FA content in 29 cold-pressed camelina (*Camelina sativa*) oils using the HH as a nutritional quality index. A relatively high HH was reported, ranging from 11.7 to 14.7, with a low IA and IT contributing to a decrease in the incidence of CHD [62]. 

Detailed information about the literature related to the HH is shown in Table 5. For shellfish, the HH value ranges from 1.73 to 4.75, except for *Loxechinus albus*. It is possible that the main food source of *Loxechinus albus* is algae, leading to a high proportion of SFA, so its HH is only 0.21, lower than in other species [34]. For fish, the value ranges from 1.54 to 4.83, with the exception of *Opisthonema oglinum,* which has an HH value of 0.87 [36]. For meat and dairy products, the ranges are 1.27–2.786, 0.32–1.29, respectively.

### 2.5. Health-Promoting Index (HPI) 

The health-promoting index (HPI) was proposed by Chen et al. in 2004 to assess the nutritional value of dietary fat [94], which focuses on the effect of FA composition on CVD. The formula is:

(4) HPI = ΣUFA/[C12:0+(4× C14:0)+ C16:0].

The HPI is the inverse of the IA. It is currently mainly used in research on dairy products such as milk [92,93,94] and cheese [57,94,95]. Detailed information about the literature related to the HPI is provided in Table 6. Its values range from 0.16 to 0.68. Dairy products with a high HPI value are assumed to be more beneficial to human health. The HPI has the same shortcoming as the IA, and it requires reliable evidence to optimize the relevant coefficients.

### 2.6. Unsaturation Index (UI) 

The UI indicates the degree of unsaturation in lipids and is calculated as the sum of the percentage of each unsaturated FA multiplied by the number of double bonds within that FA [114]. The calculation formula is:

(5) UI =1×(% monoenoics)+2×(% dienoics)+3×(% trienoics)+4×(% tetraenoics)+5×(% pentaenoics)+6×(% hexaenoics)

Unlike ∑UFA and ∑PUFA, different unsaturated FAs have different weights in the UI. This index indicates the impact of highly unsaturated FA and does not ignore the impact of FAs that have a low degree of unsaturation. In general, the UI more comprehensively reflects the proportion of FA with different degrees of unsaturation in the total FA composition of a species.

The UI is commonly used to determine the composition of macroalgal FA. It can be used as a standard for judging the content of high-quality PUFA, in which macroalgae may be used as alternative sources of high-quality PUFA instead of fish or fish oil [98]. Colombo et al. used the UI to compare macroalgae in cold water with those in warm water, with a high UI value indicating a high degree of total unsaturation. Their results suggested that the fatty acids with a high degree of unsaturation in a membrane lipid can maintain fluidity at relatively low temperature [96]. 

Detailed information about the literature related to the UI is listed in Table 7. The UI value of seaweeds varies widely from 45 to 368.68, and may be closely related to their species. There is no rule at present. The disadvantage of the UI is that it only focuses on the degree of unsaturation of FAs and does not distinguish between n-6 and n-3 FA. The fatty acids in the n-6 and n-3 series have different physiological effects on the human body.

### 2.7. Sum of Eicosapentaenoic Acid and Docosahexaenoic Acid (EPA + DHA) 

EPA and DHA are n-3 long-chain PUFAs that play essential roles in biological processes in the human body. They can reduce the risk of CVD, hypertension, and inflammation. DHA is a critical component of the retina and the neuronal system and is involved in visual functioning and cognitive functioning in humans [115,116]. The American Heart Association summarized the preventive effect of n-3 PUFA from seafood on CVD in the 2015–2020 Dietary Guidelines for Americans [1]. 

EPA and DHA can be synthesized from α-linolenic acid in the human body, but exogenous supplementation is still needed when insufficient. α-linolenic acid (ALA; C18:3 n-3) can be converted to EPA and DHA by desaturase and elongase, respectively. EPA and DHA can be supplemented by ingesting ALA. Burdge et al. [115] studied the capacity of humans to convert ALA to EPA and DHA. In a carbon isotope labeling experiment, six young male subjects orally received ^13^C-ALA as a part of their habitual diet. The results indicated that the subjects had a limited capacity to convert ALA to EPA, and ^13^C-labeling of DHA was not detected [115]. Brenna et al. summarized related studies and reached a similar conclusion [117]. Although the conversion of ALA to EPA and DHA was observed in tracer studies in all age groups, regardless of whether the study participant was male or female, the efficiency of directly supplementing with EPA to increase the level of EPA was found to be 15-fold that of supplementing with high levels of ALA. The conversion rate of ALA to DHA in infants is only 1%, and is even lower in adults [117]. Therefore, the rate of conversion of ALA to EPA and DHA that is required for health is far from sufficient; direct intake of EPA and DHA is more effective. 

EPA + DHA is an index that is recognized worldwide. Recommendations for EPA + DHA intake can be found in various dietary guidelines. According to the Food and Agriculture Organization of the United Nations (UN FAO), the recommended amount is 0.250–2 g/day. Due to the low EPA and DHA contents in terrestrial plants and animals, this index is mostly used to evaluate the nutritional value of seafood and seafood products, particularly fish, which makes it an important nutritional index for seafood. Rincón-Cervera et al. studied the fatty acid composition of fish and shellfish captured in the South Pacific, and the results showed that EPA + DHA ranged between 115.15 and 1370.67 mg/100 g in all studied fish species and between 63.61 and 522.68 mg/100 g in all studied shellfish species [34]. Detailed information about the literature related to EPA + DHA is shown in Table 8. The species of fish and shellfish as well as their nutrition intake are key factors influencing the EPA + DHA value.

### 2.8. Fish Lipid Quality/Flesh Lipid Quality (FLQ)

FLQ was originally use for fish lipid quality [107,108] or flesh lipid quality [65,66,73]. The purpose of FLQ is similar to that of the EPA + DHA index, but it calculates the sum of EPA and DHA as a percentage of total fatty acids. The formula is:

(6) FLQ =100×(C22:6 n−3+ C20:5 n−3)/ΣFA

FLQ is more suitable for marine products given their higher proportions of EPA and DHA. This index may be considered a supplement to EPA + DHA since the absolute quantity for EPA and DHA is more important. Until now, FLQ has only been used to assess the quality of lipids in fish. Senso et al. examined the fatty acid profile of the fillet of farmed sea bream (*Sparus aurata*) harvested in different seasons using FLQ as the lipid quality index. FLQ was lowest in April [73]. Detailed information about the literature related to FLQ is provided in Table 9. The value ranges from 13.01 to 36.37 for closely related species.

### 2.9. The Linoleic Acid/α-Linolenic Acid (LA/ALA) Ratio 

The linoleic acid (LA, C18:2 n-6)/α-linolenic acid (ALA, C18:3 n-3) ratio was developed for guiding infant formula. LA and ALA compete for the same desaturase and elongase enzymes, which they use to synthesize long-chain unsaturated fatty acids. Due to the low conversion rate of ALA, reducing the LA/ALA ratio only provides a modest improvement in the levels of some n-3 long-chain PUFAs; however, the balance may be the most important factor when long-chain PUFAs are not present in infant formulas. 

The Definitions & Nutrient Composition section of the Guidelines for Infant Formula published by Food Standards Australia New Zealand (FSANZ) sets the minimum and maximum proportions of LA and ALA, and specifies an LA/ALA ratio within 5:1–15:1.

The LA/ALA ratio has a higher reference value when judging the nutritional value of baby food and infant formula. Tissues of adults have a lower rate of synthesis of n-3 long-chain PUFAs than those of infants, so the LA/ALA ratio in the diet does not have too much of an impact on adults. In the literature we reviewed, the LA/ALA ratio was used in research on ruminants and dairy products as well [43,55,109]. Majdoub-Mathlouthi et al. compared the meat fatty acid composition of Barbarine lambs raised on rangelands and those reared indoors. The results showed that the grazing lambs had lower LA/ALA [43]. Sharma et al. compared the fatty acid profile of indigenous Indian cow milk with exotic and crossbred counterparts. LA/ALA was used to reflect the quality of milk [109]. LA/ALA in indigenous cattle was found to be lower than others, providing scientific data for the superiority of indigenous cow milk [109]. Detailed information of the literature related to LA/ALA is listed in Table 10. Turcana dairy ewe milk has a low LA/ALA value due to the high content of ALA given the inclusion of hemp seed in the diet [55].

### 2.10. Trans Fatty Acid (TFA) 

Most unsaturated FA in the human diet have a *cis* configuration. However, *trans* fatty acid (TFA) is present in the human diet as well. According to the Food and Drug Administration (FDA), TFA is defined as the sum of all unsaturated fatty acids that contain one or more isolated (i.e., non-conjugated) double bond(s) in a *trans* configuration [118,119]. The European Food Safety Authority (EFSA) gives a different definition of TFA, which are also present as either *trans*-MUFA or *trans*-PUFA. *Trans*-PUFAs have at least one *trans* double bond and may therefore also have double bonds in the *cis* configuration. Conjugated fatty acid (CLA) is separated from TFA as an independent section by the EFSA. CLAs may have health benefits that are different from those of TFAs, such as anti-cancer [120,121] and anti-atherosclerosis [122] activities, so it is appropriate to exclude CLA from the definition of TFA.

According to the EFSA, TFAs may originate from various sources, including of bacterial conversion of unsaturated fatty acids in the rumen of ruminants, industrial hydrogenation (used to produce semi-liquid and solid fats; can be used to produce margarine, shortening, biscuits, etc.), deodorization of unsaturated vegetable oils (or occasionally fish oils) with a high content of polyunsaturated fatty acids (a necessary step of refining), and heating and frying oil at excessively high temperatures (>220 °C) [123]. 

TFA does not play a positive role in any vital functions. On the contrary, the intake of TFA may harm human health. Evidence suggests that ruminant-derived TFA has similar adverse effects on blood lipids and lipoproteins as TFA from industrial sources. Sufficient evidence is still needed to reveal whether a difference exists between equivalent amounts of ruminant and industrially produced TFA in terms of risk of CHD [123]. *Trans*-MUFA is the most common TFA in the human diet. A few clinical trials with normotensive subjects proved that *trans*-MUFA from hydrogenated oil has no effect on systolic or diastolic blood pressure [124]. Prospective cohort studies showed that a consistent relationship exists between higher TFA intake and increased risk of CHD. Conversely, a daily intake of 3.6 g of TFA from milk fat for five weeks did not affect blood pressure or isobaric arterial elasticity [125].

According to population nutrient intake goals from the World Health Organization (WHO)/FAO, the intake of TFA should constitute less than 1% of total energy. For pregnancy and lactation, the lowest possible intake of industrially-produced TFAs is required. According to the EFSA, TFA in the diet is provided by several sources that contain essential FAs and other nutrients [124], Therefore, the EFSA panel concluded that the intake of TFA should be sufficiently reduced within a nutritionally adequate diet to lower the intake of TFA while ensuring the nutrient intake [124]. The 2015–2020 Dietary Guidelines for Americans emphasize that individuals should reduce their intake of *trans* fatty acid to as low as possible by limiting their consumption of foods that contain synthetic sources of *trans* fats. There is no need to eliminate meat and dairy products that contain small quantities of natural TFA from the diet. In the United Kingdom, the recommended intake of TFA is less than 2% of total daily energy or 5 g/day.

The TFA index is currently used in seaweed [90], lamb [45], milk [78], fish [35], and plant oil [32,33,110]. Skałecki et al. compared the fatty acid profiles of Prussian Carp fish (*Carassius gibelio*) fillets with and without skin; the share of TFA was the same in both types [35]. Mishra and Sharma monitored the changes occurring in rice bran oil and its blend with sunflower oil during repeated frying cycles of potato chips with different moisture contents (0.5% and 64.77%) [110]. The results showed that blended oil was better when used to fry dried potato chips, as TFA was the lowest after deep fat frying (increased from 1.15% to 1.80%) [110]. Detailed information of the literature related to TFA is listed in Table 11.

## 3. Conclusions

In this review, we summarized 10 FA indices that have been commonly used in the literature to characterized FA composition. Among them, PUFA/SFA, IA, IT, HH, HPI, and UI are the most frequently used indices and are widely used to evaluate a variety of research materials, mainly related to CVH. PUFA/SFA is a basic index that simply considers ∑PUFA and ∑SFA. IA, IT, HH, HPI, and UI were derived based on revising PUFA/SFA, which consider the contribution of different molecular species of SFA, as well as MUFA. However, all of these six indices do not reflect the influence of different molecular species of PUFA. For instance, n-3 PUFA and n-6 PUFA exhibit different effects on CVH. EPA + DHA and FLQ are used in the analysis of fish or shellfish, which are rich in n-3 PUFA. The LA/ALA ratio is an important index for baby food and infant formula. TFA is an indicator of food safety because it has a negative effect on many vital functions. Due to the lack of systematic integration of clinical evidence and literature data related to FA, suggesting ideas and proposals for the update of indices is difficult. Besides, CVH is the main assessment of FA indices used at present. As FA functions continue to be revealed, more indices that can be used for other diseases are expected. 

With the present review, we aimed to help researchers evaluate the nutritional value of FAs and to explore their potential usage in disease prevention and treatment, and to help newcomers to the field of FA analysis to quickly and accurately select appropriate indices. The human body is complex, so a reasonable selection of indices can help researchers to more comprehensively evaluate the research materials. The purpose of using an index is only to assess the potential nutritional and/or medicinal value of the research materials; they should not be considered gold standards. The indices should not be used indiscriminately, and the results obtained with the indices should be interpreted with caution. After a reasonable assessment using the indices, a more systematic and complex research process should be used to reach a conclusion about the nutritional effect of the research object on the human body. We recommend that researchers apply these indices to help compare several research objects to select one or more objects of interest for further in-depth research.

## Figures and Tables

**Table 1:** Summary of nutritional indices.

No.	Index	Full Name	Calculation Formula	Application
1	PUFA/SFA	Polyunsaturated fatty acid/saturated fatty acid ratio	ΣPUFA/ΣSFA	Seaweeds [27,28,29], crops [30,31], plant oil [32,33], shellfish [34], fish [34,35,36,37,38,39,40], meat [41,42,43,44,45,46,47,48,49,50,51,52,53], and dairy products [54,55,56,57]
2	IA	Index of atherogenicity	[C12:0 + (4 × C14:0) + C16:0]/ΣUFA	Seaweeds [27,28,29,58,59], crops [30,31,60,61], plant oil [33,62], shellfish [63], shrimp [64], fish [36,37,38,39,65,66,67,68,69,70,71,72,73], meat [41,42,43,48,49,50,52,53,74,75,76,77], and dairy products [54,55,56,78,79,80,81,82,83,84,85,86,87,88,89]
3	IT	Index of thrombogenicity	(C14:0 + C16:0 + C18:0)/[(0.5 ×ΣMUFA) + (0.5 ×Σn-6 PUFA) + (3 ×Σn-3 PUFA) + (n-3/n-6)]	Seaweeds [27,28,29,58,59], crops [30,31,60,61], plant oil [62], shellfish [63], shrimp [64], fish [36,37,38,39,65,66,67,68,70,71,72,73], meat [43,48,50,52,53,75,77], and dairy products [54,55,78,80,86,87,88,89]
4	HH	Hypocholesterolemic /hypercholesterolemic ratio	(cis-C18:1 + ΣPUFA)/(C12:0 + C14:0 + C16:0)	Seaweeds [90], plant oil [62], shellfish [34], fish [34,36,37,38,39,72], meat [46,47,48,52,77,91], and dairy products [54,55,78,86,87]
5	HPI	Health-promoting index	ΣUFA/[C12:0+(4 × C14:0) + C16:0]	Milk [92,93,94] and cheese [57,94,95]
6	UI	Unsaturation index	1 × (% monoenoics) + 2 × (% dienoics) + 3 × (% trienoics) + 4 × (% tetraenoics) + 5 × (% pentaenoics) + 6 × (% hexaenoics)	Seaweeds [27,28,29,59,96,97,98], crops [61,99,100], meat [44,101], and milk [102]
7	EPA + DHA	Sum of eicosapentaenoic acid and docosahexaenoic acid	C22:6 n-3 + C20:5 n-3	Shellfish [34] and fish [34,36,37,40,68,103,104,105,106]
8	FLQ	Fish lipid quality/flesh Lipid quality	100 × (C22:6 n-3 + C20:5 n-3)/ΣFA	Fish [65,66,73,107,108]
9	LA/ALA	Linoleic acid /α-linolenic acid ratio	C18:2 n-6/C18:3 n-3	Lamb [43] and milk [55,109]
10	TFA	*Trans* fatty acid	ΣTFA	Seaweeds [90], plant oil [32,33,110], fish [35], lamb [45], and milk [78]

**Table 2:** Application of PUFA/SFA in fatty acid evaluation *.

Materials		PUFA/SFA Value	Reference
Red seaweed	*Amphiora anceps*	0.42	[27]
	*Kappaphycus alvarezii*	0.57	[27]
	*Gelidiella acerosa*	0.84	[27]
	*Gelidium micropterum*	0.30	[27]
	*Gracilaria changii*	6.96 ± 0.98	[28]
	*Gracilaria corticata*	2.12	[27]
	*Gracilaria dura*	1.89	[27]
	*Gracilaria debilis*	1.17	[27]
	*Gracilaria fergusonii*	0.58	[27]
	*Gracilaria salicornia*	0.14	[27]
	*Laurencia cruciata*	0.79	[27]
	*Sarconema filiforme*	1.71	[27]
Brown seaweed	*Cystoseira indica*	1.17	[27]
	*Padina tetrastromatica*	0.85	[27]
	*Sargassum fusiforme*	0.67 ± 0.31	[29]
	*Sargassum horneri*	0.56 ± 0.06	[29]
	*Sargassum pallidum*	0.20 ± 0.09	[29]
	*Sargassum swartzii*	1.15	[27]
	*Sargassum tenerrimum*	1.18	[27]
	*Sargassum thunbergii*	0.39 ± 0.05	[29]
	*Spatoglossum asperum*	1.38	[27]
Green seaweed	*Caulerpa racemosa*	0.44	[27]
	*Caulerpa scalpeliformis*	0.88	[27]
	*Caulerpa veravalnensis*	0.73	[27]
	*Ulva fasciata*	0.42	[27]
	*Ulva reticulata*	0.23	[27]
	*Ulva rigida*	0.33	[27]
Crops	*Cyamopsis tetragonolobaL.*	1.71	[31]
	*Lupinus albus*	1.53–1.97	[30]
Plant oil	Palm stearin	0.13	[32]
	Sunflower oil	4.75–4.94	[32,33]
Shellfish	*Cancer edwardsi*	2.10	[34]
	*Cervimunida johni*	1.81	[34]
	*Concholepas concholepas*	1.16	[34]
	*Heterocarpus reedi*	1.47	[34]
	*Loxechinus albus*	0.20	[34]
	*Mesodesma donacium*	1.34	[34]
	*Pleuroncodes monodon*	1.68	[34]
	*Pyura chilensis*	1.31	[34]
	*Venus antiqua*	1.06	[34]
Fish	*Carassius gibelio*	1.62–1.70	[35]
	*Cilus gilberti*	1.15	[34]
	*Genypterus chilensis*	1.60	[34]
	*Hemiramphus brasiliensis*	1.09	[36]
	*Hyporhamphus unifasciatus*	1.11	[36]
	*Kutum roach*	1.02–1.79	[37]
	*Lagocephalus guentheri*	1.3	[38]
	*Merluccius gayi*	1.52	[34]
	*Opisthonema oglinum*	1.47	[36]
	*Orechromis niloticus*	0.51–0.56	[39]
	*Pinguipes chilensis*	0.80	[34]
	*Scomber japonicus*	0.92	[34]
	*Scomberomorus cavalla*	1.18	[36]
	*Seriola lalandi*	0.92	[34]
	*Seriolella violacea*	0.95	[34]
	*Trachinotus carolinus*	0.5–1.1	[40]
	*Trachurus murphyi*	0.95	[34]
Meat	Chicken (Caribro Vishal)	0.308–2.042	[50]
	Chicken (purchased from a hatchery and poultry farm)	0.926–0.945	[48]
	Pig (DanBred × PIC terminal line)	0.46–0.48	[49]
	Pig (Pietrain × (Duroc × Landrace))	0.85–1.29	[44]
	Lamb (Barbarine lamb)	0.13–0.37	[43,45]
	Steer (Blonded Aquitaine steer)	0.29–0.58	[42]
	Calve (75% Charolais breeds)	0.13–0.34	[51]
	Cattle (Nellore cattle)	0.11–0.20	[46]
	Yak (*Phoephagus grunniens*)	0.37–0.55	[41]
	Foal (Galician Mountain × Hispano-Bretón)	0.44–1.06	[52]
	Spanish dry-cured ham	0.19–0.30	[47]
	Bologna sausages	0.27–1.17	[53]
Dairy products	Cheese of Comisana ewe	0.086–0.173	[57]
	Milk of Chios sheep	0.06–0.08	[54]
	Milk of Karagouniko sheep	0.06–0.09	[54]
	Milk of Turcana dairy ewe	0.106–0.175	[55]
	Milk of Friesian × Jersey cow	0.02–0.04	[56]

PUFA/SFA: polyunsaturated fatty acid/saturated fatty acid ratio; * literature from 2000 until April/2020.

**Table 3:** Application of IA in fatty acid evaluation *.

Materials		IA Value	Reference
Red seaweed	*Amphiora anceps*	1.52	[27]
	*Ceramium virgatum*	0.37 ± 0.027	[59]
	*Corallina officinalis*	0.48 ± 0.039	[59]
	*Gelidiella acerosa*	0.80	[27]
	*Gelidium micropterum*	1.61	[27]
	*Gracilaria changii*	0.03 ± 0.003	[28]
	*Gracilaria corticata*	0.38	[27]
	*Gracilaria debilis*	0.69	[27]
	*Gracilaria dura*	0.45	[27]
	*Gracilaria fergusonii*	1.34	[27]
	*Gracilaria salicornia*	2.87	[27]
	*Hymenena* sp.	3.58	[59]
	*Kappaphycus alvarezii*	0.77	[27]
	*Laurencia cruciata*	0.84	[27]
	*Lomentaria clavellosa*	3.06 ± 0.611	[59]
	*Polysiphonia* sp.	1.35 ± 0.206	[59]
	*Sarconema filiforme*	0.49	[27]
Brown seaweed	*Cystoseira indica*	0.66	[27]
	*Dictyota dichotoma*	0.29 ± 0.041	[59]
	*Laminaria ochroleuca*	1.18–1.57	[58]
	*Leathesia difformis*	0.48 ± 0.021	[59]
	*Myriogloea major*	0.21 ± 0.019	[59]
	*Padina tetrastromatica*	0.81	[27]
	*Sargassum fusiforme*	0.94 ± 0.28	[29]
	*Sargassum horneri*	1.06 ± 0.06	[29]
	*Sargassum pallidum*	1.99 ± 0.45	[29]
	*Sargassum swartzii*	0.61	[27]
	*Sargassum tenerrimum*	0.66	[27]
	*Sargassum thunbergii*	1.16 ± 0.10	[29]
	*Spatoglossum asperum*	0.53	[27]
	*Undaria pinnatifida*	0.17–0.35	[59]
Green seaweed	*Caulerpa racemosa*	1.61	[27]
	*Caulerpa scalpeliformis*	0.86	[27]
	*Caulerpa veravalnensis*	1.17	[27]
	*Cladophora falklandica*	0.50 ± 0.062	[59]
	*Codium decorticatum*	0.22 ± 0.002	[59]
	*Codium fragile*	0.29 ± 0.020	[59]
	*Codium vermilara*	0.40 ± 0.086	[59]
	*Ulva fasciata*	1.37	[27]
	*Ulva reticulata*	1.54	[27]
	*Ulva rigida*	1.22	[27]
	*Ulva* sp.1	0.20 ± 0.055	[59]
	*Ulva* sp.2	0.08 ± 0.004	[59]
Crops	Cumin (*Cuminum cyminum*)	0.46–0.53	[61] ^a^
	Guar seed (*Cyamopsis tetragonoloba*)	0.22	[31]
	White lupine (*Lupinus albus*)	0.084–0.107	[30]
	*Scabiosa stellata*	0.55	[60]
Plant oil	Camelina oil (*Camelina sativa*)	0.05–0.07	[62]
	Sunflower oil	0.09–0.11	[33]
Shellfish	*Chlamys farreri*	0.31–0.37	[63]
	*Patinopecten yessoensis*	0.29–0.35	[63]
Shrimp	*Penaeus notialis*	0.71–0.82	[64]
Fish	*Abramis brama*	0.37–0.42	[65,66]
	*Clupea harengus*	0.70 ± 0.10	[66]
	*Cynoscion parvipinnis*	1.07–1.16	[67]
	*Cyprinus carpio*	0.36 ± 0.03	[66]
	*Dicentrarchus labrax*	0.40–0.42	[68]
	*Esox lucius*	0.43	[65]
	*Hemiramphus brasiliensis*	0.26	[36]
	*Hyporhamphus unifasciatus*	0.26	[36]
	*Kutum roach*	0.58–1.41	[37]
	*Lagocephalus guentheri*	0.43	[38]
	*Leuciscus idus*	0.36 ± 0.02	[66]
	*Limousin steers*	0.70–1.14	[69]
	*Micropterus salmoides*	0.29–0.68	[70]
	*Mugil cephalus*	0.91–1.22	[71]
	*Oncorhynchus mykiss*	0.33 ± 0.01	[66]
	*Opisthonema oglinum*	0.60	[36]
	*Oreochromis niloticus*	0.55–0.60	[39]
	*Perca fluviatilis*	0.37–0.44	[65,66]
	*Platichthys flesus*	0.41 ± 0.03	[66]
	*Rutilus rutilus*	0.40	[65]
	*Salmo trutta*	0.64–0.72	[72]
	*Scomberomorus cavalla*	0.48	[36]
	*Sparus aurata*	0.21–0.29	[73]
Meat	Chicken (Caribro Vishal)	0.165–0.634	[50]
	Chicken (purchased from a hatchery and poultry farm)	0.372–0.390	[48]
	Rabbit (Curcuma longa)	0.55–0.69	[75]
	Pig (DanBred × PIC terminal line)	0.27–0.31	[49]
	Lamb (Barbarine lamb)	0.49–0.52	[43]
	Lamb (Gentile di Puglia × Sopravissana)	0.99–1.32	[76]
	Lamb (Ile de France × Pagliarola)	0.71–1.06	[76]
	Lamb (Iranian fat-tailed breed)	0.53–0.77	[74]
	Heifer (Limousin heifer)	0.50–0.57	[77]
	Steer (Blonded Aquitaine steer)	0.51–0.63	[42]
	Yak (Phoephagus grunniens)	0.37–0.43	[41]
	Foal (Galician Mountain × Hispano-Bretón)	0.59–0.62	[52]
	Bologna sausages	0.33–0.60	[53]
Dairy products	Cheese of Churra ewe	1.61–3.61	[79]
	Cheese of Holstein cow	2.38–3.72	[88]
	Cheese of Italian Friesian and Italian Red Pied cattle (Caciocavallo cheese)	2.43–2.94	[84]
	Curd of cow (Middle Rhodopes)	1.94–5.02	[78]
	Milk of Anglo-Nubian goat	1.89–2.48	[81]
	Milk of goat (market of Sardinia)	2.27–2.91	[89]
	Milk of Nubian goat	1.91–2.32	[82]
	Milk of Saanen goat	2.77 ± 0.08	[85]
	Milk of Swedish Landrace goat	2.47 ± 0.07	[85]
	Milk of Chios sheep	2.00–2.72	[54]
	Milk of Karagouniko sheep	1.76–2.57	[54]
	Milk of Churra ewe	1.71–3.39	[79]
	Milk of Lacaune ewe	1.94–2.53	[80]
	Milk of Turcana dairy ewe	1.42–1.95	[55]
	Milk of cow (Middle Rhodopes)	1.88–4.18	[78]
	Milk of Friesian × Jersey cow	4.08–5.13	[56]
	Milk of Holstein cow	1.83–2.63	[88]
	Milk of Holstein–Friesian cow	1.60–3.79	[83,109] ^a^
	Milk of indigenous Indian cow	1.37	[109] ^a^
	Milk of Jersey cow	2.4823–3.4360	[87]
	Milk of Sahiwal cow	2.01	[109] ^a^
	Milk of Sahiwal × Holstein–Friesian cow	3.14	[109] ^a^
	Milk of Italian Friesian and Italian Red Pied cattle	2.49–2.99	[84]
	Yogurt of cow milk (market of Faisalabad)	1.48–2.74	[86]
	Yogurt of sheep milk (market of Faisalabad)	1.42–2.31	[86]

IA: index of atherogenicity; * literature from 2000 until April/2020; ^a^ recalculated according to the original data in the reference.

**Table 4:** Application of IT in fatty acid evaluation *.

Materials		IT Value	Reference
Red seaweed	*Amphiora anceps*	2.07	[27]
	*Ceramium virgatum*	0.12 ± 0.005	[59]
	*Corallina officinalis*	0.28 ± 0.045	[59]
	*Gelidiella acerosa*	0.52	[27]
	*Gelidium micropterum*	1.83	[27]
	*Gracilaria changii*	0.04 ± 0.01	[28]
	*Gracilaria corticata*	0.63	[27]
	*Gracilaria debilis*	1.25	[27]
	*Gracilaria dura*	0.88	[27]
	*Gracilaria fergusonii*	2.66	[27]
	*Gracilaria salicornia*	5.75	[27]
	*Hymenena* sp	2.66	[59]
	*Kappaphycus alvarezii*	1.17	[27]
	*Laurencia cruciata*	0.71	[27]
	*Lomentaria clavellosa*	2.94 ± 1.000	[59]
	*Polysiphonia* sp	0.61 ± 0.114	[59]
	*Sarconema filiforme*	0.55	[27]
Brown seaweed	*Cystoseira indica*	0.87	[27]
	*Dictyota dichotoma*	0.09 ± 0.013	[59]
	*Laminaria ochroleuca*	1.06–1.89	[58]
	*Leathesia difformis*	0.14 ± 0.006	[59]
	*Myriogloea major*	0.09 ± 0.006	[59]
	*Padina tetrastromatica*	1.20	[27]
	*Sargassum fusiforme*	0.46 ± 0.21	[29]
	*Sargassum horneri*	0.65 ± 0.07	[29]
	*Sargassum pallidum*	1.60 ± 0.56	[29]
	*Sargassum swartzii*	0.75	[27]
	*Sargassum tenerrimum*	0.90	[27]
	*Sargassum thunbergii*	0.76 ± 0.14	[29]
	*Spatoglossum asperum*	0.50	[27]
	*Undaria pinnatifida*	0.08–0.26	[59]
Green seaweed	*Caulerpa racemosa*	1.50	[27]
	*Caulerpa scalpeliformis*	1.38	[27]
	*Caulerpa veravalnensis*	1.28	[27]
	*Cladophora falklandica*	0.16 ± 0.048	[59]
	*Codium decorticatum*	0.12 ± 0.002	[59]
	*Codium fragile*	0.14 ± 0.013	[59]
	*Codium vermilara*	0.30 ± 0.080	[59]
	*Ulva fasciata*	1.56	[27]
	*Ulva reticulata*	2.90	[27]
	*Ulva rigida*	1.78	[27]
	*Ulva* sp.1	0.09 ± 0.028	[59]
	*Ulva* sp.2	0.04 ± 0.002	[59]
Crops	Cumin (*Cuminum cyminum*)	0.46–0.56	[61] ^a^
	Guar seed (*Cyamopsis tetragonoloba*)	0.53	[31]
	*Scabiosa stellata*	0.23	[60]
	White lupine (*Lupinus albus*)	0.139–0.180	[30]
Plant oil	Camelina oil (*Camelina sativa*)	0.1	[62]
Shellfish	*Chlamys farreri*	0.13–0.17	[63]
	*Patinopecten yessoensis*	0.09–0.15	[63]
Shrimp	*Penaeus notialis*	0.21–0.30	[64]
Fish	*Abramis brama*	0.23–0.24	[65,66]
	*Clupea harengus*	0.26 ± 0.04	[66]
	*Cynoscion parvipinnis*	0.18–0.29	[67]
	*Cyprinus carpio*	0.31 ± 0.03	[66]
	*Dicentrarchus labrax*	0.191–0.63	[68]
	*Esox lucius*	0.18	[65]
	*Hemiramphus brasiliensis*	0.21	[36]
	*Hyporhamphus unifasciatus*	0.44	[36]
	*Kutum roach*	0.16–0.24	[37]
	*Lagocephalus guentheri*	0.29	[38]
	*Leuciscus idus*	0.22 ± 0.05	[66]
	*Micropterus salmoides*	0.31–0.53	[70]
	*Mugil cephalus*	0.43–0.58	[71]
	*Oncorhynchus mykiss*	0.16 ± 0.01	[66]
	*Opisthonema oglinum*	0.20	[36]
	*Oreochromis niloticus*	0.82–0.87	[39]
	*Perca fluviatilis*	0.20–0.21	[65,66]
	*Platichthys flesus*	0.22 ± 0.02	[66]
	*Rutilus rutilus*	0.21	[65]
	*Salmo trutta*	0.21–0.30	[72]
	*Scomberomorus cavalla*	0.24	[36]
	*Sparus aurata*	0.14–0.19	[73]
Meat	Chicken (purchased from a hatchery and poultry farm)	0.755–0.784	[48]
	Chicken (Caribro Vishal)	0.288–1.694	[50]
	Rabbit (Curcuma longa)	0.83–1.12	[75]
	Lamb (Barbarine lamb)	1.1–1.15	[43]
	Heifer (Limousin heifer)	1.10–1.34	[77]
	Foal (Galician Mountain × Hispano-Bretón)	0.44–0.80	[52]
	Bologna sausages	0.39–1.55	[53]
Dairy products	Cheese of Holstein cow	3.22–5.04	[88]
	Curd of cow (Middle Rhodopes)	2.02–4.35	[78]
	Milk of goat (market of Sardinia)	2.70–3.20	[89]
	Milk of Chios sheep	1.24–1.46	[54]
	Milk of Karagouniko sheep	1.00–1.47	[54]
	Milk of Lacaune ewe	2.20–2.72	[80]
	Milk of Turcana dairy ewe	1.22–1.76	[55]
	Milk of Holstein cow	2.23–2.90	[88]
	Milk of Jersey cow	3.9813–4.6558	[87]
	Milk of cow (Middle Rhodopes)	2.05–4.03	[78]
	Yogurt of cow milk (market of Faisalabad)	0.39–1.84	[86]
	Yogurt of sheep milk (market of Faisalabad)	0.65–1.68	[86]

IT: index of thrombogenicity; * literature from 2000 until April/2020; ^a^ recalculated according to the original data in the reference.

**Table 5:** Application of HH in fatty acid evaluation *.

Materials		HH Value	Reference
Red seaweed	*Gelidium microdon*	4.22	[90]
	*Pterocladiella capillacea*	2.09	[90]
Brown seaweed	*Ulva compressa*	1.90	[90]
	*Ulva rigida*	1.26	[90]
Plant oil	Camelina oil (*Camelina sativa*)	11.2–15.0	[62]
Shellfish	*Cancer edwardsi*	4.75	[34]
	*Cervimunida johni*	3.48	[34]
	*Concholepas*	2.52	[34]
	*Heterocarpus reedi*	2.91	[34]
	*Loxechinus albus*	0.21	[34]
	*Mesodesma donacium*	2.15	[34]
	*Pleuroncodes monodon*	3.68	[34]
	*Pyura chilensis*	1.73	[34]
	*Venus antiqua*	1.90	[34]
Fish	*Cilus gilberti*	1.86	[34]
	*Genypterus chilensis*	2.93	[34]
	*Hemiramphus brasiliensis*	2.46	[36]
	*Hyporhamphus unifasciatus*	2.43	[36]
	*Kutum roach*	2.04–4.83	[37]
	*Lagocephalus guentheri*	2.68	[38]
	*Merluccius gayi*	2.23	[34]
	*Opisthonema oglinum*	0.87	[36]
	*Oreochromis niloticus*	1.56–1.63	[39]
	*Pinguipes chilensis*	1.54	[34]
	*Salmo trutta*	1.88–2.16	[72]
	*Scomber japonicus*	2.00	[34]
	*Scomberomorus cavalla*	1.56	[36]
	*Seriola lalandi*	2.14	[34]
	*Seriolella violacea*	2.10	[34]
	*Trachurus murphyi*	1.73	[34]
Meat	Chicken (purchased from a hatchery and poultry farm)	2.658–2.786	[48]
	Lamb (Merino Branco)	1.92	[91]
	Lamb (Ile de France × Merino Branco)	2.01	[91]
	Cattle (Nellore cattle)	1.56–2.08	[46]
	Heifer (Limousin heifer)	1.27–1.87	[77]
	Foal (Galician Mountain × Hispano-Bretón)	1.76–1.98	[52]
	Spanish dry-cured ham	2.0–2.67	[47]
Dairy products	Curd of cow (Middle Rhodopes)	0.32–0.74	[78]
	Milk of Chios sheep	0.50–0.61	[54]
	Milk of Karagouniko sheep	0.50–0.68	[54]
	Milk of Turcana dairy ewe	0.88–1.29	[55]
	Milk of cow (Middle Rhodopes)	0.34–0.75	[78]
	Milk of Jersey cow	0.4067–0.5732	[87]
	Yogurt of cow milk (market of Faisalabad)	0.54–1.12	[86]
	Yogurt of sheep milk (market of Faisalabad)	0.82–1.29	[86]

HH: hypocholesterolemic /hypercholesterolemic ratio; * literature from 2000 until April/2020.

**Table 6:** Application of HPI in fatty acid evaluation *.

Materials		HPI Value	Reference
Dairy products	Butter of Holstein cow	0.37–0.66	[93,94]
	Cheese of Red Syrian goat	0.37–0.68	[95]
	Cheese of Comisana ewe	0.42–0.50	[57]
	Cheese (Cheddar cheese) of Holstein cow	0.29–0.46	[94]
	Cheese (Provolone Cheese) of Holstein cow	0.38–0.63	[94]
	Cream of Holstein cow	0.31–0.62	[94]
	Milk of ewe (Comisana breed)	0.16–0.28	[92]
	Yogurt of Holstein cow	0.30–0.62	[94]

HPI: health-promoting index; * literature from 2000 until April/2020.

**Table 7:** Application of UI in fatty acid evaluation *.

Materials		UI Value	Reference
Red seaweed	*Ahnfeltia plicata*	250 ± 1.01	[98]
	*Amphiora anceps*	98.01, 97.5	[27,98]
	*Callophylis* sp	117	[96]
	*Ceramium virgatum*	284 ± 7	[59]
	*Corallina officinalis*	202 ± 19	[59]
	*Gelidiella acerosa*	191.02	[27]
	*Gelidium micropterum*	98.80	[27]
	*Gloiopeltis furcata*	54	[96]
	*Gracilaria changii*	368.68 ± 20.01	[28]
	*Gracilaria corticata*	257.07	[27]
	*Gracilaria debilis*	204.85, 205 ± 3.07	[27,98]
	*Gracilaria dura*	249.10, 249 ± 3.66	[27,98]
	*Gracilaria fergusonii*	134.75, 135 ± 1.14	[27,98]
	*Gracilaria salicornia*	50.631	[27]
	*Grateloupia indica*	286 ± 5.91	[98]
	*Grateloupia wattii*	181 ± 3.77	[98]
	*Hymenena* sp	45	[59]
	*Hypnea esperi*	93.6 ± 4.63	[98]
	*Hypnea musciformis*	91.3 ± 4.11	[98]
	*Kappaphycus alvarezii*	140.94, 141 ± 4.05	[27,98]
	*Laurencia cruciata*	172.95, 173 ± 5.64	[27,98]
	*Laurencia papillosa*	213 ± 4.89	[98]
	*Lomentaria clavellosa*	76 ± 12	[59]
	*Polysiphonia* sp.	143 ± 15	[59]
	*Sarconema filiforme*	245.54, 246 ± 1.27	[27,98]
	*Soliera robusta*	77	[96]
Brown seaweed	*Cystoseira indica*	195.44, 195 ± 4.21	[27,98]
	*Dictyota dichotoma*	321 ± 10	[59]
	*Leathesia difformis*	272 ± 6	[59]
	*Myriogloea major*	266 ± 7	[59]
	*Padina tetrastromatica*	154.49, 155 ± 5.50	[27,98]
	*Sargassum fusiforme*	125.65 ± 32.25	[29]
	*Sargassum horneri*	116.16 ± 5.77	[29]
	*Sargassum pallidum*	62.27 ± 15.05	[29]
	*Sargassum swartzii*	182.02	[27]
	*Sargassum tenerrimum*	187.05, 187 ± 4.47	[27,98]
	*Sargassum thunbergii*	89.87 ± 7.44	[29]
	*Spatoglossum asperum*	202.83, 203 ± 3.06	[27,98]
	*Stoechospermum marginatum*	176 ± 3.56	[98]
	*Undaria pinnatifida*	260-318	[59]
Green seaweed	*Caulerpa racemosa*	106.70, 107 ± 5.67	[27,98]
	*Caulerpa scalpeliformis*	121.67	[27]
	*Caulerpa veravalnensis*	141.87, 142 ± 2.96	[27,98]
	*Cladophora falklandica*	215 ± 7	[59]
	*Codium decorticatum*	219 ± 2	[59]
	*Codium fragile*	179 ± 11	[59]
	*Codium vermilara*	135 ± 16	[59]
	*Ulva fasciata*	102.92, 103 ± 2.83	[27,98]
	*Ulva lactuca*	87.5 ± 5.76	[98]
	*Ulva linza*	124 ± 4.23	[98]
	*Ulva reticulata*	70.87, 70.9 ± 5.33	[27,98]
	*Ulva rigida*	93.96, 93.8 ± 5.31	[27,98]
	*Ulva tubulosa*	99.6 ± 3.23	[98]
	*Ulva* sp.	76.3 ± 5.40	[98]
	*Ulva* sp.1	209 ± 20	[59]
	*Ulva* sp.2	288 ± 10	[59]
Crops	Cumin (*Cuminum cyminum*)	125.21–133.10	[61]
	Soybean (*Glycine max*)	148–155	[99]
Meat	Pig (Pietrain × (Duroc × Landrace))	111–124	[44]
	Dry-cured ham (Landrace × Large White (25% Pietrain) pig)	73 ± 6	[101]
Dairy products	Milk of (New Zealand × California) white rabbit	86–120	[102]

UI: unsaturation index; * literature from 2000 until April/2020.

**Table 8:** Application of EPA + DHA in fatty acid evaluation *.

Materials		EPA + DHA Value	Reference
Shellfish	*Cancer edwardsi*	205.62 ± 6.19 mg/100 g	[34]
	*Cervimunida johni*	162.90 ± 2.83 mg/100 g	[34]
	*Concholepas concholepas*	63.61 ± 0.42 mg/100 g	[34]
	*Heterocarpus reedi*	186.98 ± 3.88 mg/100 g	[34]
	*Loxechinus albus*	208.55 ± 10.28 mg/100 g	[34]
	*Mesodesma donacium*	216.96 ± 9.76 mg/100 g	[34]
	*Pleuroncodes monodon*	189.83 ± 3.74 mg/100 g	[34]
	*Pyura chilensis*	522.68 ± 28.02 mg/100 g	[34]
	*Venus antiqua*	214.34 ± 7.52 mg/100 g	[34]
Fish	*Cilus gilberti*	294.57 ± 8.76 mg/100 g	[34]
	*Dicentrarchus labrax*	270–480 mg/100 g	[68]
	*Genypterus chilensis*	115.15 ± 6.16 mg/100 g	[34]
	*Kutum roach*	96–250 mg/100 g	[61] ^a^
	*Merluccius gayi gayi*	309.38 ± 6.81 mg/100 g	[34]
	*Pinguipes chilensis*	507.60 ± 25.32 mg/100 g	[34]
	*Scomber japonicus*	1370.67 ± 55.79 mg/100 g	[34]
	*Seriola lalandi*	915.76 ± 19.68 mg/100 g	[34]
	*Seriolella violacea*	304.04 ± 14.15 mg/100 g	[34]
	*Trachinotus carolinus*	621–941 mg/100 g	[40]
	*Trachurus murphyi*	786.90 ± 11.44 mg/100 g	[34]
	*Epinephelus coioides*	19.9–25.4%	[103]
	*Hemiramphus brasiliensis*	16.71% ± 0.07%	[36]
	*Hyporhamphus unifasciatus*	15.53% ± 0.07%	[36]
	*Megalobrama amblycephala*	5.52–7.36%	[106]
	*Opisthonema oglinum*	40.86% ± 0.07%	[36]
	*Salmo salar*	11.80–11.81%	[104]
	*Scomberomorus cavalla*	35.06% ± 0.07%	[36]
	*Sparidentex hasta*	45.8–230.4 mg/g lipid	[105]

EPA + DHA: sum of eicosapentaenoic acid and docosahexaenoic acid; * literature from 2000 until April/2020; ^a^ recalculated according to the original data in the reference.

**Table 9:** Application of FLQ in fatty acid evaluation *.

Materials		FLQ Value	Reference
Fish	*Abramis brama*	24.46–30.14	[65,66]
	*Clupea harengus*	13.01 ± 0.77	[66]
	*Cyprinus carpio*	13.99 ± 2.15	[66]
	*Esox Lucius*	36.37	[65]
	*Leuciscus idus*	24.32 ± 2.47	[66]
	*Oncorhynchus mykiss*	17.97 ± 2.46	[66]
	*Perca fluviatilis*	30.14–33.22	[65,66]
	*Platichthys flesus*	20.25 ± 2.30	[66]
	*Rutilus*	28.41	[65]
	*Sparus aurata*	19.35–31.27	[73]

FLQ: fish lipid quality/flesh Lipid quality; * literature from 2000 until April/2020.

**Table 10:** Application of LA/ALA in fatty acid evaluation *.

Materials		LA/ALA Value	Reference
Meat	Lamb (Barbarine lamb)	6.78–10.05	[43]
Dairy products	Milk of Turcana dairy ewe	0.98–1.36	[55]
	Milk of Sahiwal cow	3.313 ± 0.262	[109]
	Milk of Holstein–Friesian cow	3.446 ± 0.196	[109]
	Milk of Sahiwal × Holstein–Friesian cow	3.065 ± 0.093	[109]
	Milk of indigenous Indian cow	2.464 ± 0.147	[109]

LA/ALA: linoleic acid/α-linolenic acid ratio; * literature from 2000 until April/2020.

**Table 11:** Application of TFA in fatty acid evaluation *.

Materials		TFA Value	Reference
Red seaweed	*Gelidium microdon*	1.34% ± 0.20%	[90]
	*Pterocladiella capillacea*	1.47% ± 0.09%	[90]
Brown seaweed	*Ulva compressa*	7.35% ± 0.63%	[90]
	*Ulva rigida*	4.89% ± 0.26%	[90]
Plant oil	Palm stearin	0.6%	[32]
	Rice bran oil	1.27–2.91%	[110]
	Sunflower oil	0.2%, 0.84–1.71%	[32,33]
Fish	*Carassius gibelio*	1.06% ± 0.06%, 10.58–37.15 mg/100 g	[35]
Meat	Lamb (Barbarine lamb)	2.23–2.83%	[45]
Dairy products	Curd of cow (Middle Rhodopes)	340–1090 mg/100 g	[78]
	Milk of cow (Middle Rhodopes)	110–210 mg/100 g	[78]

TFA: *Trans* fatty acid; * literature from 2000 until April/2020.

## Abbreviations

ALA	α-linolenic acid
AS	Atherosclerosis
CHD	Coronary heart disease
CLA	Conjugated fatty acids
CVD	Cardiovascular disease
CVH	Cardiovascular health
DHA	Docosahexaenoic acid
EFSA	European Food Safety Authority
EPA	Eicosapentaenoic acid
FA	Fatty acid
FAO	Food and Agriculture Organization
FDA	Food and Drug Administration
FLQ	Fish lipid quality/flesh lipid quality
FSANZ	Food Standards Australia New Zealand
HH	Hypocholesterolemic/hypercholesterolemic ratio
HPI	Health-promoting index
IA	Index of atherogenicity
IT	Index of thrombogenicity
LA	Linoleic acid
LDL-C	Low-density lipoprotein cholesterol
LDLR	Low-density lipoprotein receptors
MS	Multiple sclerosis
MUFA	Monounsaturated fatty acid
PPAR-γ	Peroxisome proliferators-activated receptor-gamma
PUFA	Polyunsaturated fatty acid
SFA	Saturated fatty acid
TFA	*Trans* fatty acid
TG	Triglycerides
UFA	Unsaturated fatty acid
UI	Unsaturation index
WHO	World Health Organization
